# Gender Differences In The Therapeutic Evolution Of Major Depression during COVID-19 Pandemic

**DOI:** 10.1192/j.eurpsy.2023.1347

**Published:** 2023-07-19

**Authors:** C. Navarro-Guzmán, A. Aguilar-Latorre, A. García, M. Zamrik Vila, A. M. Yáñez, M. Gili-Planas, M. Garcia-Toro

**Affiliations:** 1 University of the Balearic Islands, Palma; 2 Institute for Health Research Aragón (IIS Aragón), Zaragoza, Spain

## Abstract

**Introduction:**

The COVID-19 pandemic has posed an enormous challenge to the mental health of the population with probable differentiated profiles for men and women, although not all studies are consistent. While women are likely to have endured greater loads of stress associated with an increased incidence of mental disorders such as depression, men have been able to abuse alcohol and other drugs more, in addition to complying with prevention recommendations to a lesser extent. As soon as the COVID pandemic began, we began a clinical trial to enhance first-line treatments with three complementary interventions with patients with Major Depression (MD), which has allowed us to analyze differences in response according to gender.

**Objectives:**

As a secondary analysis of a clinical trial, the aim of the current study was to address the relative different efficacy between genders of three psychotherapeutic approaches in the context of MD.

**Methods:**

This study was a secondary analysis of a pragmatic parallel randomized controlled clinical trial that was composed of three arms (Minimal Lifestyle Intervention, Mindfulness-Based Cognitive Therapy, and Lifestyle Modification Program). We recruited 94 individuals (24 men and 70 women) from the Primary Healthcare Centers of the Balearic Islands region in Spain who were currently experiencing an episode of MD. Descriptive and univariate analyses were used to examine between-group differences in sociodemographic and clinical data between the two genders. General Linear Modelling (specifically, repeated measures ANOVA) was performed to compare the effect of gender on the evolution of depressive symptoms (measured by BDI-II).

**Results:**

Significant between-group differences were observed for the evolution of depressive symptoms after controlling for the intervention group and age. These results suggested that being a woman was significantly related to a worse evolution of depressive symptoms. This association implies a large effect size.

**Conclusions:**

The COVID 19 pandemic has not only been able to predispose women to Depression to a greater extent, but it is also possible that it has negatively conditioned their response to antidepressant therapies compared to men. However, our data suggest the possibility that greater psychological support could help prevent this situation.

**Disclosure of Interest:**

None Declared

